# Supplementary material for: Disentangling the mechanistic role of loop-C capping in Cys-loop receptor activation
Source: Nat Commun. 2025 Nov 23;16:11585. doi: 10.1038/s41467-025-66635-6 (PMC12749857; doi:10.1038/s41467-025-66635-6)
Supplement: Supplementary file 1 — Supplementary Information [file 41467_2025_66635_MOESM1_ESM.pdf]

Disentangling the mechanistic role of loop-C capping in Cys-loop receptor activation

Gisela D. Cymes and Claudio Grosman

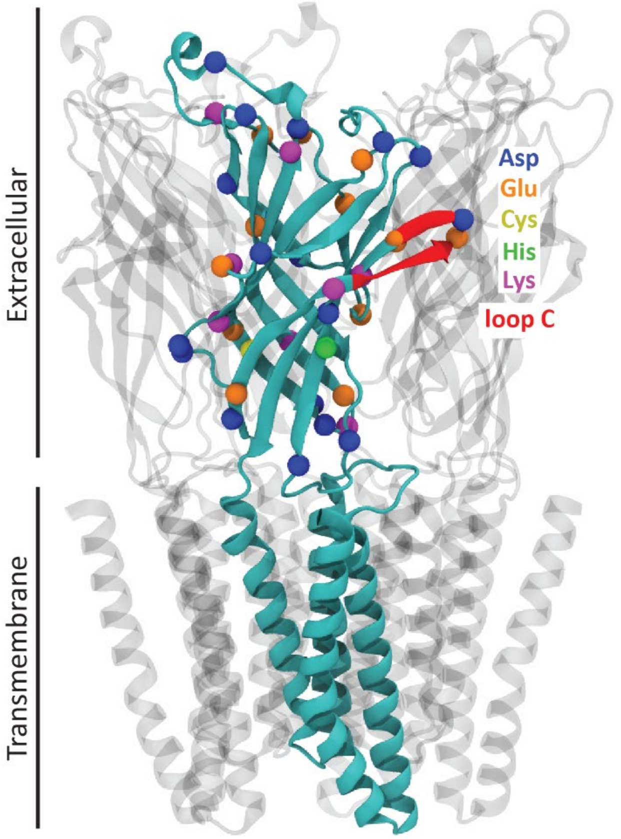

Supplementary Figure 1. Ionizable residues in the ECD of GLIC. Extracellular aspartate, glutamate, cysteine, histidine, and lysine residues mapped onto an atomic model of GLIC at pH 4.0 (PDB ID: 8WCR[1]). Owing to their high side-chain pK_a value in bulk aqueous solution, arginines were not included. The Cα atoms of extracellular ionizable residues are shown as spheres on one subunit; all other Cα atoms are shown using cartoon representation in red (loop C) or dark cyan. Although the protonation of some of these residues was deemed to be more relevant for gating than the protonation of others[2–4], it is still unclear whether liganded gating in GLIC can be ascribed to the protonation of only a few side chains. The molecular image was made with VMD[5].

Supplementary References

1. Bharambe, N. *et al.* Cryo-EM structures of prokaryotic ligand-gated ion channel GLIC provide insights into gating in a lipid environment. *Nat. Commun.* **15**, 2967 (2024).
2. Gonzalez-Gutierrez, G., Cuello, L. G., Nair, S. K. & Grosman, C. Gating of the proton-gated ion channel from *Gloeobacter violaceus* at pH 4 as revealed by X-ray crystallography. *Proc. Natl. Acad. Sci. U. S. A.* **110**, 18716–18721 (2013).

3. Nemecz, Á. *et al.* Full mutational mapping of titratable residues helps to identify proton-sensors involved in the control of channel gating in the *Gloeobacter violaceus* pentameric ligand-gated ion channel. *PLoS Biol.* **15**, e2004470 (2017).
4. Hu, H. *et al.* Electrostatics, proton sensor, and networks governing the gating transition in GLIC, a proton-gated pentameric ion channel. *Proc. Natl. Acad. Sci. U. S. A.* **115**, E12172–E12181 (2018).
5. Humphrey, W., Dalke, A. & Schulten, K. VMD: visual molecular dynamics. *J. Mol. Graph.* **14**, 33–38, 27–28 (1996).
